# Lumbar Disc Degenerative Disorder among Patients Undergoing Magnetic Resonance Imaging in a Tertiary Care Centre: A Descriptive Cross-sectional Study

**DOI:** 10.31729/jnma.7426

**Published:** 2022-05-31

**Authors:** Binit Dev, Ashutosh Shah, Aashutosh Chaudhary, Ajay Kumar Yadav, Subash Chandra Jha, Arun Kumar Chaudhary

**Affiliations:** 1 Department of Radiodiagnosis and Interventional Radiology, Birat Medical College and Teaching Hospital, Biratnagar, Morang, Nepal; 2 Kathmandu University School of Medical Sciences, Dhulikhel, Kavre, Nepal; 3 Department of Orthopedics, Birat Medical College and Teaching Hospital, Biratnagar, Morang, Nepal; 4 Department of Statistics, Nepal Commerce Campus, Tribhuwan University, Minbhawan, Kathmandu, Nepal

**Keywords:** *disc degeneration*, *low back pain*, *magnetic resonance imaging*

## Abstract

**Introduction:**

Magnetic resonance imaging is the standard imaging modality for detecting disc pathology due to its advantage of lack of radiation, multiplanar imaging capability, excellent spinal soft-tissue contrast, and precise localization of intervertebral discs changes. The aim of the study is to find out the prevalence of lumbar disc degenerative disorder among patients undergoing magnetic resonance imaging in a tertiary care hospital.

**Methods:**

A descriptive cross-sectional study was conducted in the Department of Radiodiagnosis and Interventional Radiology of a tertiary care centre among patients with complaints of low back pain from 24^th^ May, 2021 to 31^st^ December, 2021 in a tertiary care hospital. Ethical clearance was taken from the Institutional Review Committee (Reference number: 134/2077-78). A sample size of 899 was taken and convenience sampling was done. Collected data were entered and analyzed on Statistical Package for the Social Sciences version 22.0. Point estimate at 95% Confidence Interval was calculated along with frequency and percentage for binary data and mean with standard deviation for continuous data.

**Results:**

Among 899 patients undergoing magnetic resonance imaging, the prevalence of lumbar disc degenerative disorder was found to be 155 (17.24%) (14.77-19.70 at 95% Confidence Interval).

**Conclusions:**

The prevalence of lumbar disc degenerative disorder among patients undergoing magnetic resonance in our study was lower when compared to other similar studies conducted in similar settings.

## INTRODUCTION

Low back pain is the most common symptom associated with musculoskeletal spinal conditions. Discogenic pain caused by degeneration leads to changes in functional spinal motor instability.^1-4^ Magnetic Resonance Imaging (MRI) can assess neural structures, disc structures, loss of water content, vertebral body endplate signal intensity, and spondylosis of the lumbar spine.^5^

Lumbar disc degenerative disease comprises many pathologies like disc degeneration, disc displacement, Modic changes, etc.^6^ Studies regarding its prevalence are lacking in our setting.

The aim of the study was to find out the prevalence of lumbar disc degenerative disorder among patients undergoing magnetic resonance in a tertiary care hospital.

## METHODS

A descriptive cross-sectional study was conducted from 24^th^ May, 2021 to 31^st^ December, 2021 in the Department of Radiodiagnosis and Interventional Radiology at Birat Medical College after permission from the Institutional Review Committee (Reference number: IRC-PA-134/2077-78). Patients who had low back pain and had undergone MRI examination were included.

All patients presenting with low backache and with or without radiculopathy >18 years of age, patients referred from clinicians suspecting degenerative disease of the lumbar spine and patients with a lumbar degenerative disease with bowel and bladder involvement were included in the study. Patients with a history of acute trauma, surgical intervention infection, tumors and tumor-like condition and patients with contraindication to MRI such as a pacemaker, recent coronary stent, cochlear implant, and claustrophobia were excluded from the study. Informed consent was signed to indicate the patient's understanding and permission to include the patients in the study.

MRI Lumbosacral Spine (LS) spine was done on a General Electric Health-care Signa explorer 1.5T MRI scanner. MRI findings like disc desiccation, modic changes, bulge, herniation, and nerve root compression were observed.

The sample size was calculated by using the formula:

n = (Z^2^ × p × q) / e^2^

  = (1.96^2^ × 0.83 × 0.17) / 0.03^2^

  = 603

Where,

n = minimum required sample size
Z = 1.96 at 95% of Confidence Interval (CI)
p = prevalence of degenerative disease in symptomatic taken as 83%^7^
q = 1-p
e = margin of error, 3%

After adding 10% to address the non-response rate, a total sample size of 670 was obtained. However, 899 patients were enrolled in this study. Data analysis was done using the Statistical Package for the Social Sciences version 22.0. Point estimate at 95% Confidence Interval was calculated along with frequency and proportion for the binary data and mean with standard deviation for continuous data.

## RESULTS

Among 899 patients enrolled in our study, the prevalence of lumbar disc degenerative disorder among patients undergoing magnetic resonance was found to be 155 (17.24%) (14.77-19.70 at 95% Confidence Interval). On lumbar MRI overall disc desiccation (a sign of reduced disc signal intensity) being the most frequent finding was seen in 128 (82.58%) patients, followed by disc bulge 117 (75.48%), nerve root compression 117 (75.48%) patients, disc herniation 114 (73.54%). The least common finding was modic changes which were seen in 112 (72.25%).

The mean age was 47.9±15.9 years whereby 70 (45.16%) were males and 85 (54.83%) of them were females. The prevalence of lumbar desiccation findings increased with age. Disc dessication was seen in 10 (6.45%) patients aged 18-35 years, 68 (43.87%) patients aged 36-55 years and 50 (32.25%) patients aged 56-91 years. The degenerative MRI findings according to age groups are tabulated below (Table 1).

**Table 1:** Degenerative MRI findings by age (n = 155).

Age group	18-35 n (%)	36-55 n (%)	56-91 n (%)
**Disc desiccation**	10 (6.45)	68 (43.87)	50 (32.25)
**Modic change**	2 (1.29)	66 (42.58)	44 (28.38)
**Bulge**	5 (3.22)	59 (38.06)	53 (34.19)
**Herniation**	6 (3.87)	57 (36.77)	51 (32.90)
**Low back pain with radiculopathy**	5 (3.22)	59 (38.06)	53 (34.19)

The prevalence of various degenerative imaging findings was more common among females (Table 2).

**Table 2:** Degenerative MRI findings by sex (n = 155).

	Male (n = 70) n (%)	Female (n = 85) n (%)
Disc desiccation	56 (80)	72 (84.70)
Modic change	47 (67.10)	65 (76.50)
Bulge	51 (72.90)	66 (77.60)
Herniation	51 (72.90)	63 (74.10)
Low back pain with radiculopathy	51 (72.90)	66 (77.60)

Most of the degenerative findings were seen at lower lumbar levels, that is, L4/L5 and L5/S1, 35 (22.58%) and 61 (39.35%) respectively. At L5/S1 the prevalence of disc desiccation, Modic changes, disc bulge, disc herniation, and nerve root compression were 58 (37.40%), 54 (34.80%), 57 (36.70%), 53 (34.10%), and 57 (36.70%), respectively.

Nearly 94 (60.60%) of all herniated discs were protrusion. Only 3 (1.90%) discs were sequestrated. The most common location for disc herniation was posterocentral seen in 52 (33.50%) discs, followed by posterolateral and foraminal 41 (26.50%) and 15 (9.70%) respectively, so the intraspinal disc herniation (posterocentral and posterolateral) was the most common. The degenerative image findings acoording to the disc level are tabulated below (Table 3).

**Table 3:** Degenerative image findings by disc level (n = 155).

	L1-L2 n (%)	L2-L3 n (%)	L3-L4 n (%)	L4-L5 n (%)	L5-S1 n (%)
Disc desiccation	5 (3.20)	9 (5.80)	14 (9.00)	33 (21.20)	58 (37.40)
Modic change	5 (3.20)	8 (5.10)	14 (9.00)	31 (200)	54 (34.80)
Bulge	5 (3.20)	7 (4.50)	12 (7.70)	32 (20.60)	57 (36.70)
Herniation	5 (3.20)	8 (5.10)	14 (9.00)	30 (19.30)	53 (34.10)
Low back pain with radiculopathy	5 (3.20)	7 (4.50)	12 (7.70)	32 (20.60)	57 (36.70)

## DISCUSSION

Disc degeneration is loss of water content in nucleus pulposus.^8^ Modic change involves a degenerative change in the vertebral body with signal alteration of the endplate.^9,12^ Disc bulge is the presence of the disc tissue extending beyond the edges of the ring apophysis in a weakened disc, throughout the circumference of the disc in a symmetric fashion.^4,10^ Herniation is disc displacement beyond the margin of the intervertebral disc space in a localized way.^8,13,14^

Our study showed that the lumbar disc degenerative disease among patients undergoing magnetic resonance imaging was lower when compared to another similar study.^7^ The most frequent and severe changes were persistently observed at levels L4-L5 and L5-S1 irrespective of the patient's age and gender of the study participants. The occurrence at a younger age can be explained because at this level the lumbar spine is more prone to weight-bearing and repetitive minimally invasive injury. The mean age of this study group was 47.9±15.9 years; hence this further explains that degenerative changes are more common in individuals >40 years. The findings of this study were consistent with previous studies.^15^

The most observed MRI changes in our study population was disc desiccation occurring in 82.58% of cases mainly occurring at the L5-S1 level. More commonly in the middle and elderly age groups, the difference observed between the age groups was noteworthy. It did not show any gender predilection. Our findings were similar to previous studies.^16^ Modic changes were significantly noted in the middle and elderly age population in contrast to the younger age group. This can be observed due to the normal aging process. Our findings are similar to other similar studies.^17,18^

In our study, disc bulge was seen in 3.22% among 1835 years age group and 38.06% among 36-55 years age group. A number of studies have indicated that aging is an important risk factor for disc bulging. Lumbar disc degeneration, including disc dehydration and bulge and disc height narrowing, shows an increasing prevalence with age.^19^ Another study reported that increasing age correlated with a higher prevalence of disc bulge.^20^ A study showed that intervertebral discs become more convex in old age.^21^

Majority of the disc herniation was protrusion (60.6%) followed by extrusion and sequestration. Disc herniation was higher in an older population, it did not vary much in terms of gender. Various studies have reported that disc herniation is common at L4/ L5 and L5/S1. This was also reflected in this study as the majority of the herniated disc was at L4/L5 and L5/ S1, this can be due to the excessive workload causing stress at these lower lumbar levels of the spine. Disc herniation at L3/L4 and L1/L2 was observed at 9% and 3.2%, respectively, this trend is similar to previous reports. Regarding the location, the intraspinal disc herniation (postcentral and posterolateral) is the most common.^16^

Nerve root compression is most common among sciatic cases, and lower among patients with low back pain. In this study, the prevalence of nerve root compression was 36.70%. Females were slightly more affected than males, the prevalence being 77.6% and 72.9%, respectively. A study reported nerve root compression to be more frequent at level L5/S1, which is similar to this study. However, only 3.2% of patients had nerve root compression at L1/L2 level.

This study had limited interpretation because of the quality of the MRI machine as we used 1.5 Tesla MRI. Our study could have had a greater impact if we had used 3 Tesla MRIs as it has better resolution. Furthermore, a multicentered study would have given us better interpretation as our study was limited to only the centre. Also, because of the descriptive study design, causality and association could not be made among the variables

## CONCLUSIONS

This study found that the prevalence of lumbar disc degenerative disorder among patients undergoing magnetic resonance in our study was lower when compared to other similar studies. The lumbar discs are most often affected by degeneration that leads to herniation and stenosis mainly at L4-5 and L5-S1 levels, most probably because of a combination of longstanding degeneration and subsequent change in the ability of the disc to resist applied stress. All patients with degenerative disc disease presenting with low back pain with radiculopathy should undergo an MRI examination as per the literature.

Strong recommendation of MRI scans for such patients suspective of disc herniation and nerve root compression could be advised which could help in early management of this condition.
